# Cell-free fetal DNA and pregnancy-related complications (Review)

**DOI:** 10.3892/mmr.2014.3118

**Published:** 2014-12-19

**Authors:** STAVROS SIFAKIS, ZETA KOUKOU, DEMETRIOS A. SPANDIDOS

**Affiliations:** 1 Department of Obstetrics and Gynecology, University Hospital of Heraklion, Heraklion, Crete, Greece; 2 Department of Clinical Virology, School of Medicine, University of Crete, Heraklion, Crete, Greece

**Keywords:** cell-free fetal DNA, intrauterine growth restriction, non-invasive prenatal screening, obstetrics, preeclampsia, pregnancy complications, preterm labor

## Abstract

Cell-free fetal DNA (cff-DNA) is a novel promising biomarker that has been applied in various aspects of obstetrical research, notably in prenatal diagnosis and complicated pregnancies. It is easily detected by semi-quantitative PCR for the SRY target gene. It is well recognized that the levels of circulating cff-DNA play a role in various complications of pregnancy. In this review, we explore the implications of the detection of cff-DNA in a range of pregnancy-related complications, such as preeclampsia, intrauterine growth restriction (IUGR), preterm labor, placenta previa and hyperemesis gravidarum. cff-DNA is released due to apoptotic mechanisms occurring on trophoblastic cells, although recent *in vivo* studies support the existence of additional mechanisms. The increase in the levels of cff-DNA can be used to predict pregnancy-related complications and has great value in the field of prenatal diagnosis and in common pregnancy-related complications, as it precedes the clinical symptoms of the disease. Gestational age is a factor that determines the elevation in cff-DNA levels in response to pathological conditions. In conclusion, the detection of cff-DNA levels has a number of valuable applications in prenatal screening; however, the detection of cff-DNA levels has not yet been applied in clinical practice for the diagnosis of pregnancy-related disorders. Thus, studies are focusing on unraveling the etiology of alterations in its levels under pathological conditions during pregnancy, in order to determine the potenial predictive and diagnostic applications of this biomarker.

## 1. Introduction

Fetal cell circulation during pregnancy and its implications in pathological disorders is a commonly recognized factor that has been investigated in multiple studies, in contrast to the initial theory established in the early 1850s that the placenta constitutes an impenetrable barrier for the majority of proteins and chemicals between the fetus and the mother. It is well established that genetic information originating from the fetus can provide the essence for the identification and verification of genetic abnormalities, as well as highlight certain trends of pathologies associated with the mother. To this end, the application of molecular biology techniques in the non-invasive diagnosis of pregnancy-related complications has proven to be a valuable tool in genetic screening and in the evaluation of pathological conditions, as opposed to invasive techniques that require a greater economical cost and are associated with greater risks.

Cell-free fetal DNA (cff-DNA) is a molecular biomarker that has revolutionized the field of non-invasive prenatal diagnosis or screening. It was first discovered in 1997 by Lo *et al* who demonstrated the presence of fetal DNA sequences in maternal plasma and serum (1). Subsequent studies revealed that significantly greater levels of fetal DNA are present in the cell-free plasma of pregnant women compared to fetal DNA extracted from cells of the maternal blood (2). Based on these findings, further investigations have extended the detection techniques of cff-DNA by means of quantitative PCR and have focused on the clinical applications of this biomarker with particular emphasis on the prediction of pregnancy-related complications, as well as in the prenatal diagnosis or screening of fetal disorders of genetic origin (3,4). The latter approach has received considerable attention, since it has certain advantages over the isolation of initial fetal cells, such as speed, reliability, low cost and less laborious protocols (3).

**Figure 1:**
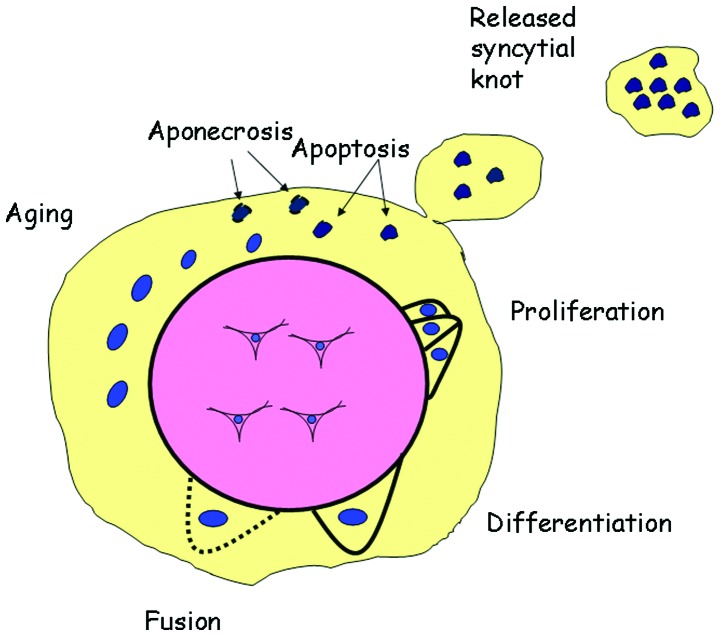
The mechanisms of trophoblast deportation into the maternal circulation. Cytotrophoblasts proliferate and differentiate during fetal development. In turn, they fuse with syncytiotrophoblasts and finally age and are packaged into apoptotic material that is deported in the maternal circulation in the form of syncytial knots. Sometimes aponecrosis occurs if the apoptotic cascade fails to take place and necrotic material is released into the maternal circulation.

The exact origin of cff-DNA remains unknown. It is suggested that it is derived mainly from the placenta, as demonstrated by the very rapid clearance of fetal DNA from maternal blood following delivery, in contrast to the majority of fetal cells that can survive several weeks post-partum (3). In addition to this evidence, the absence of circulating fetal DNA species in case reports concerning placental mosaicism confirms the origin of cff-DNA being the placenta and not the fetal unit itself (3,4). More recent studies have suggested that the major source of cff-DNA is the cells of trophoblastic origin that are released from the syncytiotrophoblast in the form of syncytial knots. These cells undergo apoptosis and the nucleic acids packed inside, including RNA and DNA are released into the maternal circulation (Fig. 1). In addition to apoptotic mechanisms taking place as a result of normal aging of the syncytiotrophoblast, accidental breakage or necrosis may also be one of the causes of the release of cell-free nucleic acids. The concept that apoptotic or aponecrotic pathways of the syncytiotrophoblast may alter the proportion of fetal DNA is gathering considerable interest (5).

In recent years, the detection of cff-DNA has been used in clinical practice for the prenatal screening of certain fetal aneuploidies, Down syndrome in particular (6). In this review, rather than focusing specifically on the applications of cff-DNA in non-invasive diagnostics, we review the implications of this biomarker in relation to pathophysiological disorders during pregrancy, such as preeclampsia, intrauterine growth restriction (IUGR) and preterm labor, commonly referred to as the ‘great obstetrical syndromes’.

## 2. cff-DNA and preeclampsia

Preeclampsia is a disorder occurring during pregnancy that is characterized by hypertension, proteinuria and edema with an onset in the second half of pregnancy. It manifests as a result of improper placentation due to impaired trophoblast differentiation. It is a leading cause of fetal mortality with a relatively high percentage of occurrence in the developed world. To date, there is no single reliable parameter used for the prediction of the development of preeclampsia, and thus, much research has focused on the discovery of novel techniques. Various studies have examined the levels of cff-DNA in pregnancies complicated by preeclamspia and have attempted to draw conclusions regarding the involvement of the latter in the development of the disease. After the discovery of cff-DNA by Lo *et al* in 1997, two years later, in a small sample size of 20 women with preeclamspia, the same group of researchers demonstrated that the levels of cff-DNA were increased by 5-fold compared to those of the normotensive controls (7). These results were confirmed by later studies, including those of Leung *et al* (8) and Zhong *et al* (9) where approximately the same level of increase in cff-DNA was reported in pregnant women with preeclamspia (8–10). More importantly, cff-DNA levels appeared to increase before the onset of the clinical symptoms of the disease. Levine *et al* followed these conclusions and conducted a larger study comprising of 120 pergnant women with preeclamspia and 120 controls, and observed a 2- to 5- fold increase in cff-DNA levels in the women with the disease (11). Moreover, a two-phase elevation in serum cff-DNA levels in the women who developed preeclampsia was observed between 17 and 28 weeks of gestation, as well as 3 weeks before the onset of clinical symptoms (11). The elevated cff-DNA levels in women with preeclampsia have been verified using a second detection technique based on the quantification of the DYS14 sequence instead of the SRY target (12). The authors revealed a greater increase in the levels of cff-DNA of up to 10-fold compared to the control subjects (12). Initially, it was thought that a combination of the increased release of DNA from the abnormal placenta and a reduced cff-DNA clearance due to impaired liver and kidney functions are likely to be responsible for the elevating cff-DNA levels (10,11).

**Figure 2:**
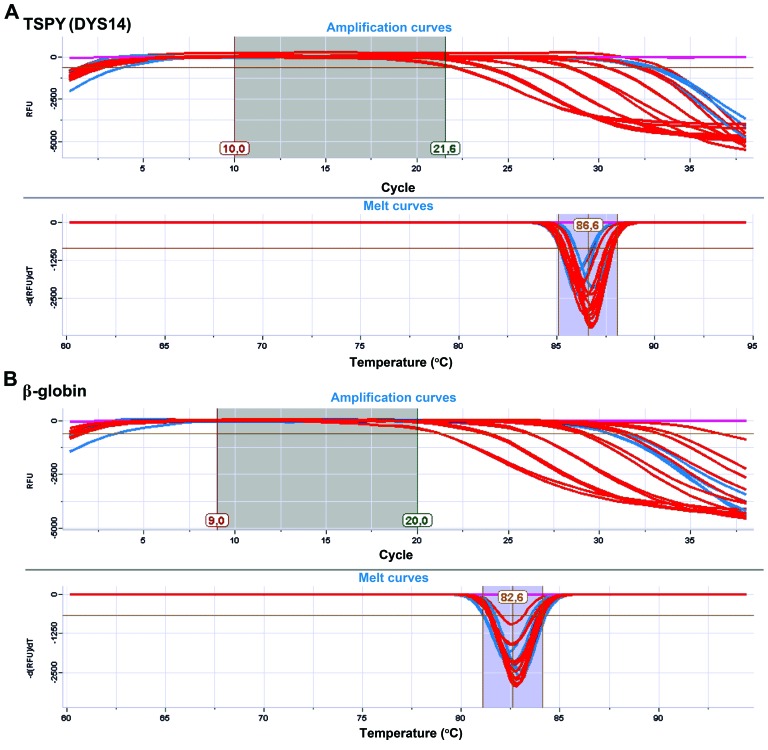
Representative amplification plots (upper panels) and melt curves (lower panels) for the markers, (A) TSPY (DYS14) and (B) β-globin. The amplification plots show the relative fluorescence units (RFU) at each cycle of the reaction. Red lines represent the standard samples (7- to 5-fold serial dilutions, ranging from 460 to 0.029 ng), and the light blue lines represent two indicative unknown samples. The negative controls (NTCs) are depicted in purple. The absence of secondary peaks in the melt plot, confirms the specificity of the primers used (courtesy of Dr A. Zaravinos).

In contrast to the data from the above studies, in 2007, Crowley *et al* demonstrated no actual difference in the levels of cff-DNA between the preeclamptic and normotensive controls by measuring the quantity of the SRY gene (13). These results were confirmed by other studies demonstrating that cff-DNA levels were not significantly altered between pregnancies complicated by preeclampsia and normal pregnancies (14,15). A previous study demonstrated that serum cff-DNA levels were higher in women with preeclampsia compared to those of the controls from 17 to 20 weeks of gestation; however, these levels did not differ significantly from those of the controls at 25–28 weeks, and no differences were observed during early pregnancy (13–16 weeks) (11). Sifakis *et al* (16) demonstrated that the levels of cff-DNA increased at an early gestational age (11–13 weeks) in pregnant women who were destined to suffer from severe preeclampsia at a later stage; notably, in women with late (mild) preeclampsia, the cff-DNA levels were similar to those noted in women with normal pregnancies (16). Moreover, the incease in cff-DNA levels was not as dramatic (95.5 vs. 51.5 genome equivalents) as that noted in other studies (8–11), as the authors utilized the more sensitive DYS14 locus technique for quantification (Fig. 2). Mechanistically, the difference in the results of the latter study may be due to improper placental perfusion that induces oxidative stress and apoptosis in the early stages before the clinical symptoms of the disease. By contrast, Levine *et al* suggested that the apoptosis of trophoblasts occurs as a secondary effect in response to hypoxia that is required for the differentiation of the placenta in the first trimester, suggesting that cff-DNA is expected to remain stable in early gestation in preeclampsia (11). In addition to this evidence, a recent report shed light on the putative mechanisms underlying the pathophysiology of cff-DNA and preeclampsia. Scharfe-Nugent *et al* suggested that cff-DNA has a pro-inflammatory effect and that the rapid elevation of the latter in pregnancy may act as a danger signal to the mother that fetal cells are dying (17). This was demonstrated in BALB/c mice and by the measurements of interleukin (IL)-6 production following the intraperitoneal administration of cff-DNA. Furthermore, this effect seemed to be Toll-like receptor-9 (TLR-9)-dependent, as TLR-9(−/−) mice were shown to be protected from fetal resorption and the induction of inflammation (17).

In general, there is conflicting evidence as regards the time point of the elevation of cff-DNA levels in preeclampsia (16,11). However, the general consensus that is supported by a large body of experimental results suggests that cff-DNA levels are increased prior to the development of the symptoms of the disease and can be used as a predictive marker for preeclampsia, at least for the severe type and early onset of the disease. The mechanisms involved are more complex than those originally believed and involve a combination of the apoptosis, hypoxia and inflammation pathways.

## 3. cff-DNA and IUGR

IUGR is a complex disorder of pregnancy with varying etiology. It is characterized by the failure of the fetus to achieve its normal growth potential and is associated with perinatal morbidity and mortality, as well as cardiovascular disease in adult life (18). Although a number of different causes have been attributed to the development of IUGR, placental dysfunction is one of the predominant causes.

The studies that have examined the occurrence of IUGR in relation to cff-DNA levels are fewer than those that have examined preelampsia and cff-DNA. A early study in 2003 by Sekizawa *et al* addressed the issue of circulating fetal DNA levels in pregnancies complicated by IUGR and found that fetal DNA levels in pregnancies complicated by IUGR were similar to those in the controls, even though only 9 IUGR and 20 control cases were examined in their study (19). A later study in 2006 by Smid *et al* demonstrated increased levels of cff-DNA in fetuses with fetal growth restriction (FGR) (20). These findings were confirmed by the more recent studies of Alberry *et al* (21), and Al Nakib *et al* (22), who used considerably higher sample sizes compared to those used by Sekizawa *et al* in 2003 (19). cff-DNA levels were shown to be significantly higher in pregnancies complicated by fetal growth restriction than in normal pregnancies (21,22), which was partially due to placental insufficiency (22).

## 4. cff-DNA and preterm labor

Preterm labor is defined as labor that begins before the 37th week of pregnancy and occurs due to contractions of the uterus, resulting in changes of the cervix that begin before 37 weeks of pregnancy. It is associated with cervical ripening and membrane rupture and is a major cause of neonatal death and long-term handicaps in infants (23). Several etiological factors have been proposed to be responsible for the development of the disease, such as the impairment of vascular-trophoblast invasion and physical stimuli (24–26).

The exploration of using cff-DNA as a predictive biomarker of placental hypoxic dysfunction disorders, including pre-term labor/delivery has received much attention over the years. The preliminary study by Hoesli *et al* in 2002 demonstrated no significant differences in erythroblast numbers between 47 pregnancies complicated by preterm contractions between 20 and 34 weeks of gestation and an equal number of matched controls (24). In 2005, Farina *et al* demonstrated that cff-DNA levels were increased in maternal serum in patients with a high risk of spontaneous preterm delivery either by preterm labor or by the premature rupture of membranes (25). On the other hand, Illanes *et al* demonstrated no significant association between the maternal plasma levels of cff-DNA at 22 to 24 weeks of pregnancy and gestational age at delivery, supporting the hypothesis that cff-DNA levels may not predict preterm labor in women with a short cervix (26). The findings of Farina *et al* are in agreement with those of a recent study, in which the levels of cff-DNA were examined as a predictor of spontaneous preterm delivery in a total sample size of 1,316 women (27). Statistically significant differences were observed between cff-DNA levels above the 95th centile assessed by routine RHD genotyping at 25 weeks of gestation and subsequent spontaneous preterm delivery with a strongest association observed for delivery before 34 weeks (27). In a smaller sample size of 60 pregnant women, including 30 patients with established preterm labor, the mean cff-DNA levels were approximately 6-fold higher in the pathological group compared with the control group (28). In addition, Quezada *et al* (29) examined the fetal fraction of cff-DNA measured at 10–19 weeks of gestation in 103 pregnancies that delivered <37 weeks, in 21 that delivered <34 weeks and in 82 that delivered between 34 and 37 weeks of gestation out of a total of 3,169 pregnancies. The authors found no significant differences between the spontaneous preterm delivery groups and the term delivery group (29). Perhaps the most conclusive report in terms of elucidating a plausible mechanism of action between preterm labor and cff-DNA levels was provided in the study by Scharfe-Nugent *et al*. In that study, the authors suggest that cff-DNA plays a pro-inflammatory role and is capable of activating nuclear factor (NF)-κB through IκB degradation, resulting in the production of pro-inflammatory IL-6 in human peripheral blood mononuclear cells (PBMCs) (17). Preterm birth was rapidly induced in mice following the administration of fetal DNA by intraperitoneal injection on gestational weeks 10–14, whereas TLR9(−/−) mice were protected from these effects, highlighting TLR9 as a potential therapeutic target for preterm birth (17).

Taking all the above data into consideration, there is contradictory evidence as to whether the elevation of cff-DNA levels in cases of preterm delivery precedes the clinical event and whether cff-DNA can be used as a surrogate marker for predictive diagnostics. It is generally accepted that cff-DNA is released as a result of the early initiation of the breakdown of the placental barrier in anticipation of labor (25). However, in most cases, the elevated levels of cff-DNA seem to be a consequence of the process that initiates the onset of labor and subsequent delivery, and not a predictive marker of the pathology itself. Consistent with this finding, the recent study by Poon *et al* demonstrated that fetal and maternal cff-DNA levels were affected by maternal characteristics, although the corrected values in 20 cases of spontaneous preterm delivery did not differ significantly from those of the 1,805 unaffected pregnancies (14).

## 5. cff-DNA and other pregnancy-related complications and pathologies

cff-DNA has been examined as a surrogate biomarker in relation to pregnancy-related pathological disorders apart from preeclampsia, IUGR or preterm birth. Vora *et al* reported a correlation between body mass index (BMI) and cff-DNA levels in 16 obese women and 14 lean women compared to 10 control pregnancies, suggesting that this may reflect increased adipocyte necrosis and stromal vascular apoptosis occurring in obese subjects (30). This notion was supported by *in vitro* experimental findings where stromal vascular cells displayed an increased protein expression of caspase-9 and caspase-3 in obese as opposed to lean subjects, suggesting that the active remodeling of adipose tissue in obese pregnant women may result in the increased release of cff-DNA into the circulation (31). cff-DNA was further explored in a study on pregnancies complicated by placental previa, and the results indicated that at 15–28 weeks of gestation, the concentration of hypermethylated RASSF14A was significantly higher in the pathological group (n=14) compared to the normal pregnancy group (n=161) (32). In addition, cff-DNA appears to play a significant role in women with hyperemesis gravidarum, as a predictor of clinical symptoms, as in a previous study, the concentrations of cff-DNA were found to be significantly higher in 16 women suffering from the disease (range, 21.6–311.2 genome equivalents/ml) compared to 23 women with normal pregnancies (range, 6.6–59.6 genome equivalents/ml) (33). The same group published a short report two years later, confirming the above-mentioned findings in a larger sample size of 157 controls and 45 pathological subjects (34). In that study, cff-DNA levels were found to be considerably higher (approximately 2.5-fold) in cases of severe hyperemesis gravidarum compared to mild and moderate states of the disease (1.26- and 1.6-fold increase) (34). The etiology for this apparent increase was thought to be the hyperactivation of the maternal immune system that is responsible for the onset of hyperemesis gravidarum, while maternal immune tolerance is being established, that in turn may result in the invasion of the myometrium by growing trophoblasts originating from the invasive placenta (34,35).

## 6. Conclusions

The discovery of fetal cells and cff-DNA has revolutionized the field of non-invasive prenatal diagnosis and has opened a new avenue in the field of obsterical research. The detection of cff-DNA with the current state of the art knowledge of quantitative PCR is easy, rapid and inexpensive, and thus provides significant advantages over older protocols involving the isolation of fetal cells. It is well established that the increase in cff-DNA levels can be used as a predictive marker for the early detection of pregnancy-related disorders, such as preeclampsia, IUGR, preterm labor, placental previa and hyperemesis gravidarum, although conflicting evidence suggests that cff-DNA levels may increase during the early stages of pathological changes and may later decrease as the disease progresses. Despite this discrepancy, cff-DNA levels clearly increase prior to the onset of the clinical symptoms of pregnancy-related complications. The mechanisms of action are believed to be a combination of apoptotic, aponecrotic and inflammatory events that manifest during placental development. Further and more intensive research is required to elucidate the exact pathways that govern the underlying pathology of the release of cff-DNA and pregnancy-related complications, although the results presented thus far indicate that cff-DNA should be considered as a serious addition to the field of non-invasive prenatal screening and the early evaluation of severe pregnancy-related complications.

## References

[1] Lo YM, Corbetta N, Chamberlain PF, Rai V, Sargent IL, Redman CW, Wainscoat JS (1997). Presence of fetal DNA in maternal plasma and serum. Lancet.

[2] Lo YM (2000). Fetal DNA in maternal plasma: biology and diagnostic applications. Clin Chem.

[3] Hahn S, Huppertz B, Holzgreve W (2005). Fetal cells and cell free fetal nucleic acids in maternal blood: new tools to study abnormal placentation?. Placenta.

[4] Masuzaki H, Miura K, Yoshiura KI, Yoshimura S, Niikawa N, Ishimaru T (2004). Detection of cell free placental DNA in maternal plasma: direct evidence from three cases of confined placental mosaicism. J Med Genet.

[5] Litton C, Stone J, Eddleman K, Lee MJ (2009). Noninvasive prenatal diagnosis: past, present, and future. Mt Sinai J Med.

[6] Sifakis S, Papantoniou N, Kappou D, Antsaklis A (2012). Noninvasive prenatal diagnosis of Down syndrome: current knowledge and novel insights. J Perinat Med.

[7] Lo YM, Leung TN, Tein MS, Sargent IL, Zhang J, Lau TK, Haines CJ, Redman CW (1999). Quantitative abnormalities of fetal DNA in maternal serum in preeclampsia. Clin Chem.

[8] Leung TN, Zhang J, Lau TK, Chan LY, Lo YM (2001). Increased maternal plasma fetal DNA concentrations in women who eventually develop preeclampsia. Clin Chem.

[9] Zhong XY, Holzgreve W, Hahn S (2002). The levels of circulatory cell free fetal DNA in maternal plasma are elevated prior to the onset of preeclampsia. Hypertens Pregnancy.

[10] Wataganara T, Bianchi DW (2004). Fetal cell-free nucleic acids in the maternal circulation: new clinical applications. Ann N Y Acad Sci.

[11] Levine RJ, Qian C, Leshane ES, Yu KF, England LJ, Schisterman EF, Wataganara T, Romero R, Bianchi DW (2004). Two-stage elevation of cell-free fetal DNA in maternal sera before onset of preeclampsia. Am J Obstet Gynecol.

[12] Zimmermann B, El-Sheikhah A, Nicolaides K, Holzgreve W, Hahn S (2005). Optimized real-time quantitative PCR measurement of male fetal DNA in maternal plasma. Clin Chem.

[13] Crowley A, Martin C, Fitzpatrick P, Sheils O, O’ Herlihy C, O’ Leary JJ, Byrne BM (2007). Free fetal DNA is not increased before 20 weeks in intrauterine growth restriction or pre-eclampsia. Prenat Diagn.

[14] Poon LC, Musci T, Song K, Syngelaki A, Nicolaides KH (2013). Maternal plasma cell-free fetal and maternal DNA at 11–13 weeks’ gestation: relation to fetal and maternal characteristics and pregnancy outcomes. Fetal Diagn Ther.

[15] Stein W, Müller S, Gutensohn K, Emons G, Legler T (2013). Cell-free fetal DNA and adverse outcome in low risk pregnancies. Eur J Obstet Gynecol Reprod Biol.

[16] Sifakis S, Zaravinos A, Maiz N, Spandidos DA, Nicolaides KH (2009). First-trimester maternal plasma cell-free fetal DNA and preeclampsia. Am J Obstet Gynecol.

[17] Scharfe-Nugent A, Corr SC, Carpenter SB, Keogh L, Doyle B, Martin C, Fitzgerald KA, Daly S, O’Leary JJ, O’Neill LA (2012). TLR9 provokes inflammation in response to fetal DNA: mechanism for fetal loss in preterm birth and preeclampsia. J Immunol.

[18] Gourvas V, Dalpa E, Konstantinidou A, Vrachnis N, Spandidos DA, Sifakis S (2012). Angiogenic factors in placentas from pregnancies complicated by fetal growth restriction (Review). Mol Med Rep.

[19] Sekizawa A, Jimbo M, Saito H, Iwasaki M, Matsuoka R, Okai T, Farina A (2003). Cell-free fetal DNA in the plasma of pregnant women with severe fetal growth restriction. Am J Obstet Gynecol.

[20] Smid M, Galbiati S, Lojacono A, Valsecchi L, Platto C, Cavoretto P, Calza S, Ferrari A, Ferrari M, Cremonesi L (2006). Correlation of fetal DNA levels in maternal plasma with Doppler status in pathological pregnancies. Prenat Diagn.

[21] Alberry MS, Maddocks DG, Hadi MA, Metawi H, Hunt LP, Abdel-Fattah SA, Avent ND, Soothill PW (2009). Quantification of cell free fetal DNA in maternal plasma in normal pregnancies and in pregnancies with placental dysfunction. Am J Obstet Gynecol.

[22] Al Nakib M, Desbriere R, Bonello N, Bretelle F, Boubli L, Gabert J, Levi-Mozziconacci A (2009). Total and fetal cell-free DNA analysis in maternal blood as markers of placental insufficiency in intrauterine growth restriction. Fetal Diagn Ther.

[23] Simons FE, Schatz M (2012). Anaphylaxis during pregnancy. J Allergy Clin Immunol.

[24] Hoesli I, Danek M, Lin D, Li Y, Hahn S, Holzgreve W (2002). Circulating erythroblasts in maternal blood are not elevated before onset of preterm labor. Obstet Gynecol.

[25] Farina A, LeShane ES, Romero R, Gomez R, Chaiworapongsa T, Rizzo N, Bianchi DW (2005). High levels of fetal cell-free DNA in maternal serum: a risk factor for spontaneous preterm delivery. Am J Obstet Gynecol.

[26] Illanes S, Gomez R, Fornes R, Figueroa-Diesel H, Schepeler M, Searovic P, Serra R, Perez A, Nien JK (2011). Free fetal DNA levels in patients at risk of preterm labour. Prenat Diagn.

[27] Jakobsen TR, Clausen FB, Rode L, Dziegiel MH, Tabor A (2012). High levels of fetal DNA are associated with increased risk of spontaneous preterm delivery. Prenat Diagn.

[28] El-Garf W, Sheba M, Salama S, Fouad R, El-Shenawy M, Bibers M, Azmy O (2013). Assesment of plasma cell-free fetal DNA using hypermethylated RASSF1A in maternal plasma in cases of spontaneous preterm labor. Gynecol Obstet.

[29] Quezada MS, Francisco C, Dumitrascu-Biris K, Nicolaides KH, Poon LC (2014). Fetal fraction of cell-free DNA in maternal plasma in the prediction of spontaneous preterm delivery. Ultrasound Obstet Gynecol.

[30] Vora NL, Johnson KL, Basu S, Catalano PM, Hauguel-De Mouzon S, Bianchi DW (2012). A multifactorial relationship exists between total circulating cell-free DNA levels and maternal BMI. Prenat Diagn.

[31] Haghiac M, Vora NL, Basu S, Johnson KL, Presley L, Bianchi DW, Hauguel-De Mouzon S (2012). Increased death of adipose cells, a path to release cell free DNA into systemic circulation of obese women. Obesity (Silver Spring).

[32] Kim MJ, Kim SY, Park SY, Ahn HK, Chung JH, Ryu HM (2013). Association of fetal derived hypermethylated RASSF1A concentration in placenta-mediated pregnancy complications. Placenta.

[33] Sekizawa A, Sugito Y, Iwasaki M, Watanabe A, Jimbo M, Hoshi S, Saito H, Okai T (2001). Cell-free fetal DNA is increased in plasma of women with hyperemesis gravidarum. Clin Chem.

[34] Sugito Y, Sekizawa A, Farina A, Yukimoto Y, Saito H, Iwasaki M, Rizzo N, Okai T (2003). Relationship between severity of hyperemesis gravidarum and fetal DNA concentration in maternal plasma. Clin Chem.

[35] Minagawa M, Narita J, Tada T, Maruyama S, Shimizu T, Bannai M (1999). Mechanisms underlying immunologic states during pregnancy: possible association of the sympathetic nervous system. Cell Immunol.

